# EEG response to game-craving according to personal preference for games

**DOI:** 10.1093/scan/nsaa131

**Published:** 2020-10-16

**Authors:** Jihyeon Ha, Wanjoo Park, Sang In Park, Chang-Hwan Im, Laehyun Kim

**Affiliations:** Center for Bionics, Korea Institute of Science and Technology, Seoul, 02792, Korea; Department of Biomedical Engineering, Hanyang University, Seoul, 04763, Korea; Engineering Division, New York University Abu Dhabi, Saadiyat Island, Abu Dhabi, 129188, United Arab Emirates; Center for Bionics, Korea Institute of Science and Technology, Seoul, 02792, Korea; Department of Biomedical Engineering, Hanyang University, Seoul, 04763, Korea; Center for Bionics, Korea Institute of Science and Technology, Seoul, 02792, Korea

**Keywords:** internet gaming disorder, craving, EEG, preference, parieto-occipital theta power

## Abstract

Recently, the World Health Organization included ‘gaming disorder’ in its latest revision of the international classification of diseases (ICD-11). Despite extensive research on internet gaming disorder (IGD), few studies have addressed game-related stimuli eliciting craving, which plays an important role in addiction. Particularly, most previous studies did not consider personal preferences in games presented to subjects as stimuli. In this study, we compared neurophysiological responses elicited for favorite game (FG) videos and non-favorite game (NFG) videos. We aimed to demonstrate neurophysiological characteristics according to the game preference in the IGD group. We measured participants’ electroencephalogram (EEG) while they watched FG, NFG and neutral videos. For FG videos, the parieto-occipital theta power (TP_PO_) were significantly increased compared with those for NFG videos (*P* < 0.05, paired *t*-test). TP_PO_ also differed significantly between the healthy control and IGD groups only on FG videos controlling covariate (TP_PO_ on neutral videos) (*P* < 0.05, analysis of covariance [ANCOVA]). And TP_PO_ was significantly correlated to self-reported craving score only on FG videos (r = 0.334, *P* < 0.05). In the present study, we demonstrate that FG videos induce higher TP_PO_ than that induced by NFG videos in the IGD group and TP_PO_ is a reliable EEG feature associated with craving for gaming.

## Introduction

Internet-related addictions have emerged as social problems, particularly for male-adolescents/adults (Young, 1998b). Many studies reported that male-adolescents tended to invest and try to spend more time for playing the game (Kuss, 2013; Spekman *et al.*, 2013; Chen *et al.*, 2018). Pre-occupation with internet games often becomes a behavioral addiction that, similar to drug addiction, deteriorates physical and mental health. Internet game addiction, among internet-related addictions, is called ‘internet gaming disorder (IGD)’ and was also included in section 3 of the diagnostic and statistical manual of mental disorder-V (DSM-V) as a behavioral addiction in 2013(American Psychiatric Association, 2013; Petry and O’Brien, 2013). Recently, the World Health Organization included ‘gaming disorder’ in its latest revision of the International Classification of Diseases (ICD-11) as a disorder due to addictive behavior (World Health Organization, 2018).

Many epidemiological studies had been conducted to determine the seriousness of IGD. However, Griffiths *et al.* (2016) mentioned that many researchers, rather than conducting further epidemiological studies, had to conduct treatment-seeking studies in the clinical population. Many studies of IGD have been conducted to demonstrate neurophysiological characteristics in various experimental paradigms, for example, resting state, task-related response and induction of craving. Particularly, craving, impaired control and continued behavioral engagement have been argued as important factors for research into not only IGD but also substance use disorders and behavioral addictions (Potenza, 2006). Therefore, craving has been demonstrated to play an important role in the perpetuation of behavioral addictions (McRae-Clark *et al.*, 2011). Because functional magnetic resonance imaging (fMRI) could precisely image the functional activity of the brain, several such neurophysiological studies for craving on IGD have been conducted. However, electroencephalogram (EEG)-based studies are lacking. Although EEG has a high temporal resolution and simplicity of measurement, and EEG-based applications are relatively suitable for diagnosis and treatment, previous studies for primitive research on, for instance, reward circuitry, have largely been fMRI-based. Currently, there is a need for EEG-based studies on craving in IGD.

Many fMRI studies had used experiments presenting game-related stimuli to induce craving. The dorsolateral prefrontal cortex, orbitofrontal cortex, anterior cingulate and parahippocampal gyrus were more activated when the IGD group was exposed to game-related pictures than when exposed to neutral pictures (Ko *et al.*, 2009, 2013). They noted that cue-induced game-related craving in those with IGD was similar to that in those with substance dependence. In further studies, they demonstrated that the fronto-limbic network, particularly the parahippocampus, played an important role in not only cue-induced smoking craving but also cue-induced game-related craving (Ko *et al.*,2013). One study found the presentation of videos induced craving more than that of pictures (Tong *et al.*, 2007). The idea of presentation of video stimuli that is particularly suitable for game-related stimuli is reliable. Several fMRI studies have presented video cues as stimuli for game-related craving. In the anterior cingulate and orbitofrontal cortex, reported as important areas for game-related craving, activity increased in these areas in the excessive internet game-playing group in response to game-related video cues (Han *et al.*, 2010). From the same experiment, they demonstrated that self-reported craving was positively correlated with the beta values of the left inferior frontal gyrus, left parahippocampal gyrus and right/left thalamus (Han *et al.*, 2015).

We reviewed many previous studies to investigate the neurophysiological features and analyze neuroimaging and stimuli for craving. Evidently, game-related craving studies are less than those investigating resting state and ERP. Currently, no standardized features, analytic methods, and stimuli to induce craving have been defined. In the case of craving studies in particular, selecting stimuli is an extremely important concern because several studies showed that the response to the stimulus depends on the preference (Kawasaki and Yamaguchi, 2012; Sawata *et al.*, 2015). In most craving studies, however, they did not consider participants’ preference when researchers selected stimuli (Dymond *et al.*, 2014; Ko *et al.*, 2013; Liu *et al.*, 2017; Phillips *et al.*, 1994; Rangaswamy *et al.*, 2003; Reid *et al.*, 2006).

Here, we considered participants’ preference on game based on the assumption that favorite game (FG) elicits stronger craving for game than non-favorite game (NFG). We aimed to demonstrate neurophysiological characteristics at the cortical level based on preference of participants toward games in the IGD group. We designed the experiment to induce craving by presenting gameplay videos, which were classified as FG and NFG videos, to each participant. Based on our experimental design, we hypothesized that the following: (i) there would be different EEG pattern according to game preferences and (ii) FG videos would induce craving more than NFG videos. In this study, we studied the following comparisons: (i) NFG and FG videos compared by paired sample *t*-test and (ii) NFG and FG videos controlling neutral videos compared by analysis of covariance (ANCOVA).

## Materials and methods

### Design

#### Selection of stimuli for inducing craving.

In a pre-online survey for selection of stimuli, we selected three types of games [FIFA online 3 (FIFA), Sudden Attack (SA) and League of Legends (LOL)] as stimuli, based on their position as the top three PC games in South Korea in 2016 (Gametrics, 2016). We selected 12 game-playing videos (12: 3 types of game videos × 4; running time of video: 5 min). All participants watched videos intended to induce craving, and thereafter, they self-reported their degree of craving. We selected two high-scoring videos per game. Each selected video was divided into six 25-s videos. We finally chose 36 game-playing videos. For the neutral videos, we selected 36 natural videos scoring medium levels of arousal and valance. The videos selected for this study are attached to this manuscript as a supplementary file (Video V1) and can also be found on https://youtu.be/K83jANLQoHE.

#### Experimental protocol.

**Fig. 1.**
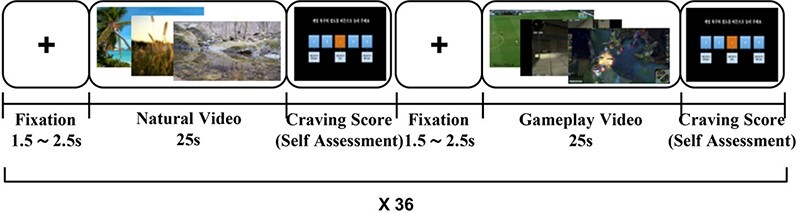
Schematic representation of the experimental protocol. In the fixation section, we presented a fixation cross during 1.5 or 2.5 s. We alternately presented neutral video and three kinds of gameplay videos: FIFA, SA and LOL. We presented 72 videos to each participant, all of which were counterbalanced. In the craving score section, after watching a video, the participants self-assessed the degree of craving they were feeling at that moment according to the 5-point Likert scale.

All participants watched 36 game-playing videos and 36 neutral videos, and alternately used a head-mounted display (HMD) device (Oculus DK2 HMD; Oculus VR LLC, Menlo Park, CA, USA) to enhance immersion. Although one study asserts that HMD did not impinge on EEG signal (Cattan *et al.*, 2018), we conducted advanced preprocessing using the artifact subspace reconstruction (ASR) method to reduce various artifacts without loss of meaningful EEG (Mullen *et al.*, 2013). Figure 1 schematically represents the experimental protocol. We used video clips showing dynamic scenes from addictive gameplay (FIFA, SA and LOL), while counterbalancing their appearance frequencies. We designed a section of natural video viewing, aimed at a wash-off or neutralizing effect after watching each trial of gameplay video. After watching each video clip, the participants self-reported the degree of craving degree that they were feeling at that moment according to the 5-point Likert scale. The self-assessment questionnaire was as follows: please press the button (1–5) of the degree of game craving that you are feeling now (1: I do not feel any craving for gaming now; 3: I feel craving for gaming now; 5: I feel very strong craving for gaming now).

### Participants

We recruited 62 adolescent/late-adolescent males (age: 19.31 ± 2.51 years) to participate in our experiment. Because there was no ‘Gold standard’ for IGD assessment, we chose the Young’s internet addiction test which has been most commonly used to evaluate the severity of game addiction (Young, 1998a). And Young’s internet addiction test was validated in Korea (Lee *et al.*, 2013). We classified all participants into the healthy control (HC) and IGD groups according to the Korea version of Young’s internet addiction test (Y-IAT-K). In this study, all participants watched three types of game-playing videos to induce game-related craving. The inclusion criteria for the IGD and HC groups were as follows: (i) For the HC group, Y-IAT-K scores < 40, and for the IGD group, Y-IAT-K scores > 60 and (ii) the participants had a FG among FIFA, SA, and LOL. The exclusion criteria for IGD and HC were as follows: (i) patients diagnosed another substance abuse; (ii) patients with current or previous episodes of neurophysiological disease and (iii) participants who preferred other games to FIFA, SA or LOL. We excluded seven participants in the EEG analysis because of a failure to record clean EEG data, and 15 participants because they did not satisfy the inclusion criteria or belonged to the exclusion criteria, and finally analyzed EEG data of 40 participants (age: 19.15 ± 2.52 years, Young scale: 47.61 ± 21.64) who satisfied the inclusion criteria. We classified 20 participants into the HC group according to their Y-IAT-K score (age: 19.00 ± 2.60 years), and 20 participants into the IGD group according to their Y-IAT-K score (age: 19.30 ± 2.49 years). Before commencing the experiment, we explained all the experimental procedures to the participants or their guardians and each participant responded to a fixed-choice questionnaire (1-FIFA, 2-SA, 3-LOL) to declare their FG. We regarded the other, unselected games on the questionnaire as NFG [Distribution of FG types: HC (FIFA 6/SA 4/LOL 10), IGD (FIFA 1/SA 3/LOL 16)]. After they had participated in the experiment, we rewarded participants with monetary remuneration. This experimental study was approved and reviewed by the Institutional Review Board (IRB) [Approval number: 2017–013] of the Korea Institute of Science and Technology (KIST).

### Measurements and processing

**Fig. 2.**
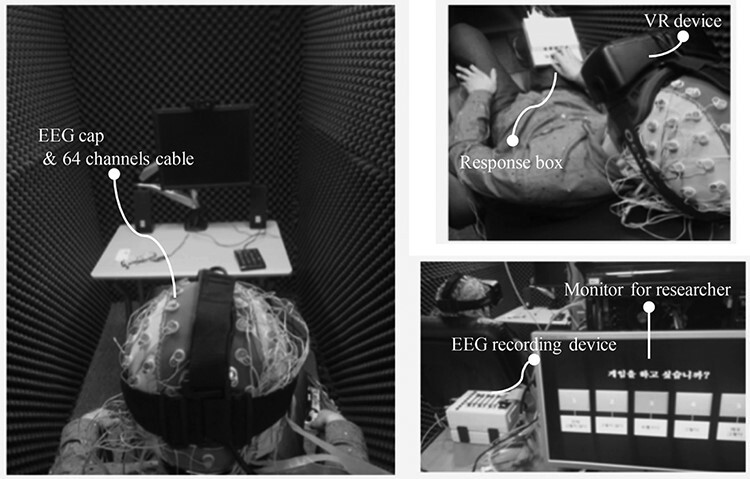
Experimental setup.

An EEG recording system (sampling rate: 2048 Hz; Active-two, Biosemi S.V., Amsterdam, Netherlands) was used. EEG signals were acquired using a cap providing 64 electrodes positioned according to the International 10/20 system. Figure 2 shows EEG recording system with HMD. EEG data preprocessing and analysis were conducted by using EEGlab (http://sccn.ucsd.edu/eeglab), a toolbox of MATLAB (2018b, Mathworks Inc., Natick, MA, USA). To pre-process the EEG data, we first down-sampled the EEG data to 512 Hz and epoched it during the watching of video clips. We conducted 0.5- to 50-Hz band-pass filtering on the epoched EEG data and then removed eye movement and muscle artifacts from the data by conducting ASR (Mullen *et al.*, 2013). Finally, we re-referenced the data by using the common average reference. We used EEG relative power as features. Relative power is the percentage of power in any band compared with the total power in the EEG (for instance, ‘relative theta’ is the percentage of theta of the combined sum of delta, theta, alpha, beta and gamma). We calculated the power spectral density (PSD) of the EEG using Welch’s method for calculating EEG power (Stoica and Moses, 2005) and five band power using PSD: delta (1–4 Hz), theta (4–8 Hz), alpha (8–12 Hz), beta (12–30 Hz) and gamma (30–50 Hz) (Kim *et al.*, 2013; Roh *et al.*, 2016). We denoted regions of interest for EEG analysis as prefrontal, central and parieto-occipital areas. In this study, EEG power of the prefrontal area means the average of channels in the prefrontal area (Fpz, Fp1, AFz and Fp2). Similarly, EEG power of the central area means the average of channels in the central area (C3, C1, Cz, C2 and C4), and EEG power of the parieto-occipital area means the average of channels in the parieto-occipital area (POz, Oz, PO3 and PO4).

### Statistical analysis

We used the Shapiro–Wilk test (SW-test) to test the normality of our dataset. Based on the SW-test, all our demographic and EEG datasets satisfied normality. We conducted the Chi-square test to confirm matched ages between the HC and IGD groups and a two-sample *t*-test to compare demographic data (game playtime, Y-IAT-K) between the HC and IGD groups. We used the paired sample *t*-test to study within-subjects’ statistics for craving score and within-subjects’ EEG power while they watched NFG and FG videos, and ANCOVA, a technique for analyzing grouped data having covariate (in this study, EEG power while participants watched neutral videos) to study between-subjects’ statistics for EEG power in the HC and IGD groups while they watched NFG and FG videos (Keselman *et al.*, 1998). Partial correlation was used to analyze the correlation between self-reported craving score and EEG features on game-play videos controlling two covariates (self-reported craving score and EEG features on neutral videos) (Fisher, 1924). We adjusted our statistical significance by performing the Bonferroni correction because we had 12 indicators [areas of interest (prefrontal, central and parieto-occipital areas) × frequency band of interest (relative delta, theta, alpha and beta power)]. For our two hypotheses, we obtained significant *P*-values of 0.041 and 0.0083 (significant EEG power, *P* = 0.05/12 and *P* = 0.01/12). Here, we denoted the significant *P*-values *P* < 0.041 and *P* < 0.0083 as *P* < 0.05 and *P* < 0.01, adjusted by the Bonferroni correction, in the statistical results for EEG. We calculated the expected effect size for the two-sample *t*-test, paired sample *t*-test (Cohen’s d) and ANCOVA (partial eta-squared, $\eta$*_p_*^2^) using G*power (Faul *et al.*, 2007 2009). In the case of Cohen’s d, standard values of 0.10, 0.25 and 0.40 for effect size are generally considered small, medium and large, respectively. And expected effect sizes for Cohen’s d in this study are 0.909 (two-sample *t*-test) and 0.660 (paired sample *t*-test). In the case of partial eta-squared value ($\eta$*_p_*^2^), standard values of 0.01, 0.06 and 0.14 for effect size are generally adjudged small, moderate and large, respectively. And expected effect sizes for partial eta-squared value ($\eta$*_p_*^2^) is 0.171. We performed the SW-test, two sample *t*-test, paired sample *t*-test and partial correlation using GraphPad Prism (Version 8.00 for MAC, GraphPad Software, La Jolla California USA) and ANCOVA using the statistical toolbox of MATLAB (2018b, Mathworks Inc., Natick, MA, USA).

## Results

### Demographic data

We present the demographical data of participants in Table 1. We found no significant differences in age between the HC and IGD groups (*P* = 0.744). The Y-IAT-K scores of the IGD group were significantly higher than those of the HC group (*P* < 0.001), with a large effect size (Cohen’s d 1.62). Similarly, the average time of gameplay per day in the previous week for the IGD group was significantly longer than that of the HC group (*P* < 0.001), with a large effect size (Cohen’s d 8.35).

**Table 1.** Mean, s.d. and P-value of chi-square test and two samples t-test on demographic data

	Mean (s.d.)		
	HC group	IGD group	*P*
**Age**	19.00 (2.60)	19.30 (2.49)	0.744
**Game playtime**	1.00 (1.48)	7.95 (5.87)	<0.001^***^
**Y-IAT-K**	25.70 (5.32)	68.15 (4.84)	<0.001^***^

*** Significant *P*-value for two samples’ *t*-test.

### Self-reported craving score

**Fig. 3.**
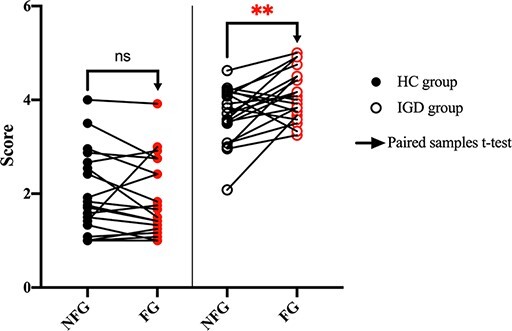
Analysis of the self-reported craving scores of 40 participants. Left: paired plot of mean craving scores after presentation of NFG and FG videos in the HC group [NFG mean (s.d.): 1.97 (0.874), FG mean (s.d.): 1.88 (0.839)]; right: paired plot of mean craving scores after presentation of NFG and FG videos in the IGD group [NFG mean (s.d.): 3.66 (0.595), FG mean (s.d.): 4.10 (0.535)]. We indicate the scores of each participant after presentation of NFG videos using black circles and the scores after presentation of FG videos using red circles. Filled circles indicate the HC group and empty circles indicate the IGD group. Two paired rightward arrows in the HC and IGD groups indicate that we conducted a paired sample *t*-test between NFG and FG videos. (*, **) represent (*P* < 0.05, *P* < 0.01), respectively.

Figure 3 shows the paired plots of mean craving scores after presentation of NFG and FG videos to the HC and IGD groups. In the IGD group, the mean craving score after showing FG videos was significantly higher than that after showing NFG videos, with a large effect size [IGD: *P* < 0.01 (Cohen’s d 0.788)]. However, these scores did not significantly differ in the HC group [HC: *P* > 0.05].

### EEG spectral analysis

#### Comparison within subjects’ EEG power during exposure to NFG and FG videos.

**Fig. 4.**
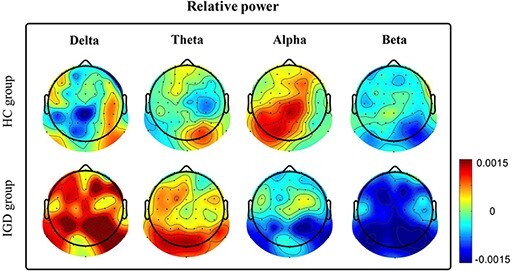
Topographies of difference between EEG power (frequency bands of interest: delta, theta, alpha and beta) during exposure to NFG and FG videos in the HC and IGD groups [difference: EEG relative power during exposure to FG videos–EEG relative power during exposure to NFG videos]. Right: colorbar illustrates the relationship between the colors of the colormap and the difference between levels of EEG power (colorbar scale: −0.0015 to 0.0015). If the EEG power exposed to FG videos is higher than that to NFG videos, topographic colors are close to red. In the opposite case, topographic colors are close to blue.

**Fig. 5.**
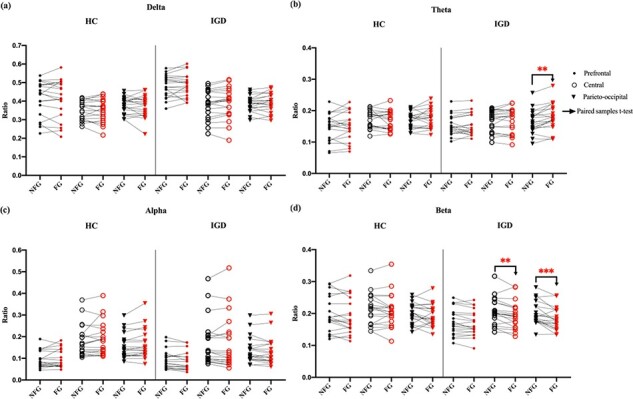
Analysis of the EEG power of the IGD group according to brain area classified as three areas (frequency bands of interest: delta, theta, alpha and beta; brain areas of interest: prefrontal, central and parieto-occipital). (a) Paired plot of delta power during exposure to NFG and FG videos; (b) paired plot of theta power; (c) paired plot of alpha power; (d) paired plot of beta power; we indicate the EEG powers of each participant while they watched NFG videos in black and those of each participant while they watched FG videos in red. Filled circles indicate the prefrontal area, empty circles indicate the central area, and inverted triangles indicate the parieto-occipital area. Paired rightward arrows indicate that we conducted paired sample *t*-test between EEG powers while the IGD group watched NFG and FG videos, in each frequency band for each area. (ns, *, **, ***) represents (*P* > 0.05, *P* < 0.05, *P* < 0.05 adjusted by the Bonferroni correction, *P* < 0.01 adjusted by the Bonferroni correction), respectively.

In Figure 4, we show the scalp topography of differences in EEG powers during exposure to NFG and FG videos in the HC and IGD groups. In Figure 5, we show a plot of paired lines between EEG powers during exposure to NFG and FG videos, according to the frequency band. We did not draw the plot for the HC group, because the EEG powers of the HC group did not significantly differ over any area. As shown in Figures 4 and 5, the theta power in the parieto-occipital area and beta power in the central and parieto-occipital area were statistically strongly paired only in the IGD group. The theta power during exposure to FG videos was significantly increased compared with that for NFG videos [parieto-occipital area: *P* < 0.05 adjusted by the Bonferroni correction, with a large effect size (Cohen’s d 1.23)]. The beta power during exposure to FG videos was significant decreased compared with that for NFG videos [central area: *P* < 0.05 adjusted by the Bonferroni correction, with a large effect size (Cohen’s d 1.14); parieto-occipital area: *P* < 0.01 adjusted by the Bonferroni correction, with a large effect size (Cohen’s d 1.27)]. However, the delta and alpha power in the IGD group did not significantly differ over the entire brain area and the HC group for all frequency bands over the whole brain area because the channels showing significant variations in Figure 4 did not include regions of interest.

#### Comparison between subjects’ EEG power during exposure to neutral and game (NFG and FG) videos.

**Fig. 6.**
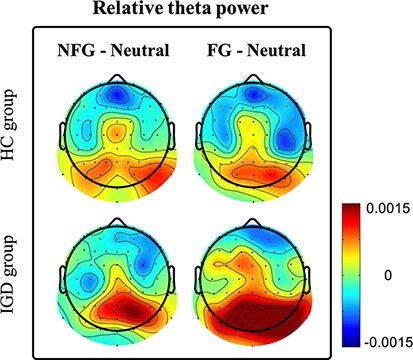
Topographies of differences between TP_PO_ during exposure to game videos [NFG and FG] and neutral videos in HC and IGD groups [difference: EEG relative power during exposure to NFG or FG videos–EEG relative power during exposure to neutral videos]. Right colorbar illustrates the relationship between the colors of the colormap and the difference between our EEG powers (colorbar scale: − 0.0015–0.0015). The higher the EEG power during exposure to game videos than that for neutral videos, the closer the topographic colors are to red. In the opposite case, topographic colors are close to blue.

**Fig. 7.**
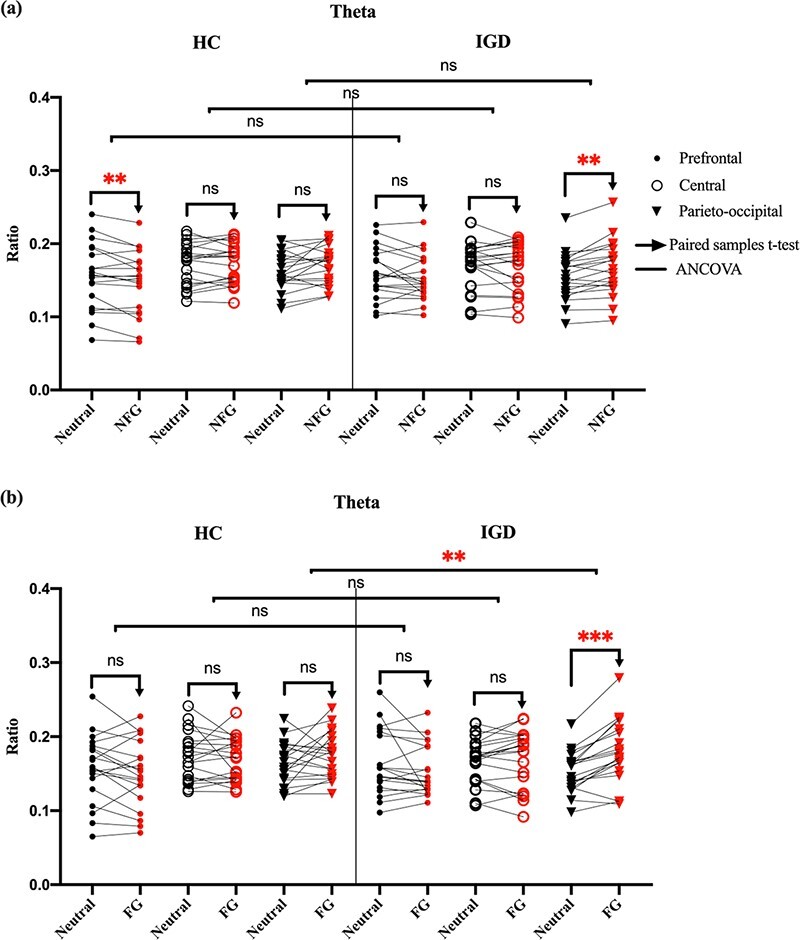
(a) Paired plot of the theta power during exposure to NFG and neutral videos in the HC and IGD groups according to brain area classified as three areas (brain area of interest: prefrontal, central, and parieto-occipital); (b) paired plot of the theta power during exposure to FG and neutral videos in the HC and IGD groups. We indicate the theta power of each participant while they watched neutral videos in black and the theta power of each participant while they watched game videos (NFG or FG) in red. Filled circles indicate the prefrontal area, empty circles indicate the central area, and inverted triangles indicate the parieto-occipital area. Paired rightward arrows indicate that we conducted paired sample *t*-tests between EEG powers while the IGD group watched neutral and game (NFG or FG) videos in each frequency band in each area. A line between the HC and IGD groups indicates that we conducted ANCOVA between the HC and IGD groups. (ns, **, ***) represent (*P* > 0.05 adjusted by the Bonferroni correction, P < 0.05 adjusted by the Bonferroni correction, *P* < 0.01 adjusted by the Bonferroni correction), respectively.

In Figure 6, we show the scalp topography of differences in EEG power during exposure to game videos (NFG and FG) and neutral videos in the HC and IGD groups. As shown in Figure 7, the theta power of the IGD group during exposure to game (NFG and FG) videos was significantly higher in the parieto-occipital area than that during exposure to neutral videos [between NFG and neutral videos: *P* < 0.05 adjusted by the Bonferroni correction, with a large effect size (Cohen’s d 1.28); between FG and neutral videos: *P* < 0.01 adjusted by the Bonferroni correction, with a large effect size (Cohen’s d 1.83)]. Conversely, the theta power of the HC group did not significantly differ over any area. In the prefrontal area, the theta power of the HC group during exposure to game (NFG and FG) videos was significantly lower than that for neutral videos [between NFG and neutral videos: *P* < 0.05 adjusted by the Bonferroni correction, with a large effect size (Cohen’s d 0.943)]. To compare the effect of NFG and FG videos on the HC and IGD groups, we conducted ANCOVA considering condition of neutral videos [comparison between theta powers during exposure to NFG and FG videos with those for neutral videos in the HC and IGD groups]. As shown in Figure 7 and Table 2, the IGD group had significantly higher parieto-occipital theta power (TP_PO_) than the HC group only watching FG videos controlling covariate [NFG, F(1,37) = 5.82, *P* > 0.05 adjusted by the Bonferroni correction; FG: F(1,37) = 10.35, *P* < 0.05 adjusted by the Bonferroni correction, with a large effect size ($\eta$*_p_*^2^ = 0.223)].

**Table 2.** ANCOVA result: comparison of theta power for neutral and game (NFG and FG) videos in HC and IGD groups

	Mean (s.d.)				
	HC group	IGD group	F	*P*	$\eta$ *_p_* ^2^
**Prefrontal**					
Neutral	0.155 (0.045)	0.158 (0.034)			
NFG	0.147 (0.043)	0.154 (0.031)	1.62	0.211	0.043
**Central**					
Neutral	0.172 (0.029)	0.170 (0.032)			
NFG	0.173 (0.027)	0.167 (0.034)	0.150	0.697	0.004
**Parieto-occipital**					
Neutral	0.163 (0.026)	0.155 (0.032)			
NFG	0.170 (0.026)	0.166 (0.038)	5.82	<0.05	0.139
**Prefrontal**					
Neutral	0.157 (0.045)	0.163 (0.043)			
FG	0.150 (0.045)	0.154 (0.034)	2.02	0.164	0.053
**Central**					
Neutral	0.172 (0.033)	0.167 (0.030)			
FG	0.169 (0.030)	0.171 (0.039)	7.60	<0.05	0.174
**Parieto-occipital**					
Neutral	0.163 (0.028)	0.152 (0.027)			
FG	0.175 (0.031)	0.179 (0.042)	10.35	<0.05^**^(bf)	0.223

** Significant *P*-value for ANCOVA; bf, adjusted by the Bonferroni correction.

### Correlation analysis between self-reported craving score and significant EEG feature

**Fig. 8.**
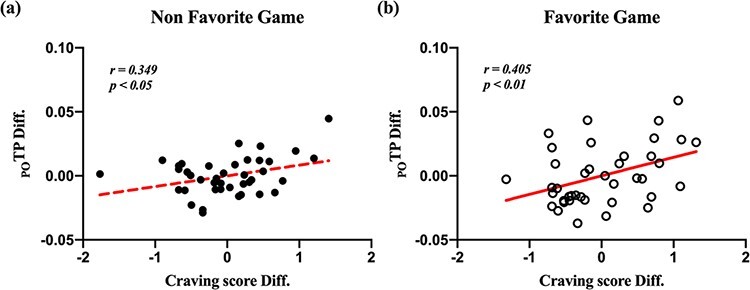
Plot for partial correlation between self-reported craving score and TP_PO_ on gameplay videos controlling self-reported craving score and TP_PO_ on neutral videos. (a) Empty black circles indicate each participant on non-favorite gameplay videos; (b) Filled black circles indicate each participant on favorite gameplay videos. ‘TP_PO_ Res.’ means that residuals between TP_PO_ on gameplay videos and predicted TP_PO_ by multiple regression considering two covariates (self-reported craving score and TP_PO_ on neutral videos). ‘Craving score Res.’ means that residuals between self-reported craving score on gameplay videos and predicted self-reported craving score by multiple regression considering two covariates (self-reported craving score and TP_PO_ on neutral videos).

In Figure 8, we show the plot for residuals of self-reported craving score and on TP_PO_ with linear regression lines. Multiple regression was conducted for partial correlation and to calculate the residuals with two covariates (Self-reported craving score and TP_PO_ on neutral videos). Correlation coefficient between self-reported craving score and TP_PO_ on FG videos controlling neutral videos is higher than NFG videos controlling two covariates by partial correlation (NFG: *r* = 0.280, *P* > 0.05; FG: *r* = 0.334, *P* < 0.05).

## Discussion

We demonstrated neurophysiological characteristics according to the preference of stimuli and found reliable EEG features of the craving state using FG videos as stimuli. We discuss four steps in this section: (i) demographic data: HC and IGD groups; (ii) self-reported craving score: NFG and FG videos (paired sample *t*-test) and (iii) EEG power analysis: NFG and FG videos with neutral videos (paired sample *t*-test, ANCOVA result and correlation between self-reported craving score and significant EEG feature).

(i) In the result of our demographic data, we verify that appropriate participants comprised the HC and IGD groups, and well-classified participants elicited distinctive results. As shown in Table 1, male adolescent/late-adolescent participants were demographically matched by age. The IGD group had not only a higher average time of gameplay per day in the previous week than that of the HC group but also a higher Y-IAT-K score because our target in this study was the IGD group rather than the recreational internet gaming use group. In this study, there is a limitation of our study in recruitment. Although both HC and IGD groups’ FG is a LOL, the number of participants who respond that FIFA or SA is their FG are relatively small.

(ii) As shown in Figure 3 for the self-reported craving score between NFG and FG videos, the craving score after exposure to FG videos was significantly higher than that for NFG videos in the IGD group. The craving score after exposure to FG videos in the HC group did not differ significantly from that for NFG videos. Therefore, we inferred that the stimuli (both NFG and FG videos) did not act as stimulants for them and that notions of FG and NFG were obscure to them. Therefore, we would expect a difference in brain activity during exposure to NFG and FG videos, particularly in the IGD group. And we presented internet gameplay videos as stimuli to induce craving unlike most studies that have presented pictures as stimuli (Ko *et al.*, 2009; Liu *et al.*, 2017; Zhang *et al.*, 2016). We predicted these types of stimuli to effectively induce craving because videos are more immersive than pictures (Tong *et al.*, 2007), particularly in game-related stimuli.

(iii) Before the interpretation of significant EEG features, we found the references from other addictions because there have been few EEG studies for IGD. According to behavioral addiction review paper (Grant *et al.*, 2010), behavioral addiction is phenomenologically similar to substance use disorder (e.g. craving state). According to IGD review papers (Young and Brand, 2017; Kuss *et al.*, 2018), the authors conclude that IGD and substance use disorder have similar neurobiological mechanism based on many previous studies especially in cue-based reactivity. In this study, we conducted cue-based experiment in order to induce game-related craving. So, we cited some studies for other addictions to interpret our main results with respect to craving.

We reported significant results decreased beta and increased theta power by comparing EEG features between watching NFG and FG videos (paired sample *t*-test). Several studies have reported a decrease in beta power associated with subjectively experienced relaxation (Ray and Cole, 1985; Knyazev, 2007). We infer that the individuals in the IGD group were relaxed and not in a stressed state when they watched FG videos as compared with in those watching NFG videos. However, no preference- or craving-related studies referred to decreased beta power. Previous studies on other addictions have reported increased EEG theta power as a craving-related feature (Phillips *et al.*, 1994; Rangaswamy *et al.*, 2003; Reid *et al.*, 2006; Dymond *et al.*, 2014). Therefore, we assert that the difference in theta power elicited by FG and NFG videos possibly reflects a difference in the craving rate. We expect that FG videos will induce a higher level of craving-related brain activity than that induced by NFG videos.

Especially, we also reported significant results, TP_PO_ by comparing three statistical tests: (i) EEG features between watching neutral and game-play videos (NFG and FG) on HC and IGD group, respectively (paired sample *t*-test); (ii) EEG features between two groups watching game-play videos controlling covariate (TP_PO_ on neutral videos, ANCOVA) and (iii) Correlation between self-reported craving score and TP_PO_ controlling two covariates (self-reported craving score and TP_PO_ on neutral videos). Based on these results, we insist that TP_PO_ is associated with craving and this craving is modulated by context memory based on game-related experience. In order to supplement the interpretation, we reviewed fMRI-based craving studies for all addictions including IGD and some EEG studies for other addictions because previous EEG-based game-related craving-inducing studies were few.

Montag and Reuter (2017) reported that hippocampus made emotional responses based on context memory when exposed to game related cue. Ko *et al.* (2013) also reported that there was strong activity of parahippocampal gyrus in state of game and smoking urge. The authors inferred that game experience (especially winning) based on memory could induce craving. And some authors reported that precuneus, parahippocampus with respect to emotion related memory for gaming cue induced craving would work respectively. As follows: Precuneus, the processing of visual stimuli and in the recollection of positive experiences such as winning (Cavanna and Trimble, 2006); parahippocampus, storing memories of the gaming experience and processing their emotional significance (Salzmann *et al.*, 1993; Rudy, 2009). Several studies based on craving cue demonstrated that cocaine (Grant *et al.*, 1996) or alcohol (Franken *et al.*, 2003) related memory is associated with craving. In order to fill a gap between fMRI- and EEG-based studies, there are also many studies that hippocampus, parahippocampal gyrus (Bennett and Gottfried, 1970; Sainsbury, 1998; Hasselmo, 2005; Moore *et al.*, 2006; Olvera-Cortés *et al.*, 2013) and precuneus (Li *et al.*, 2009; Hebscher *et al.*, 2019) are associated with theta power activation. There have been also many studies that theta power activation is associated with memory (Meltzer *et al.*, 2009; Staudigl *et al.*, 2010). According to these previous studies, the interpretation that an increase in theta power is associated with craving state and it is modulated by context memory is reliable. We also infer that these results appeared well because FG that participants mostly have experience were used as stimuli.

Particularly, we are convinced that TP_PO_, rather than features of other studies, correlates with game-related craving for IGD because our results were triple-checked by three step analysis. First, the TP_PO_ for those watching FG videos was higher than that of those watching NFG videos in the IGD group. Second, the TP_PO_ for those watching FG videos was higher than that of those watching neutral videos in the IGD group. This feature differed significantly between the IGD and HC groups. Third, the TP_PO_ was significantly correlated to self-reported craving score.

Most studies inducing craving have been limited to presenting stimuli related to types of addiction, regardless of personal preference. Previous studies on game-related craving did not compare brain activity during FG cues with that during NFG cues because they analyzed only game cues and neutral cues. Therefore, unlike previous studies, this experimental protocol elicited fascinating results. We found two important contributions beyond this study. First contribution is that preferred stimuli are more stimulative than non-preferred stimuli. In the present study, we infer that FG related memory induce craving state better than NFG. Based on previous studies, the game-related cue in itself was critical to induce craving in experiments targeted on IGD, although it was not a preferred cue. In future craving study with respect to all other addictions, the consideration of subjective preference should be among the important issues. For example, researchers of participants who prefer wine in a craving-inducing study for alcohol use disorder should use wine-related stimuli, rather than beer-related stimuli. Second contribution is that we report the increased TP_PO_ as a reliable feature in the craving state. Because there have been few EEG-based studies dealing with craving on IGD. This quantitative feature will be a helpful and suitable reference for EEG-based treatments or diagnostics targeting IGD. For example, cue-exposure therapy (CET) have been widely used for treatment over other addictions including IGD (Choi *et al.*, 2011; Lee *et al.*, 2007; Marissen *et al.*, 2007; Park *et al.*, 2015; Shin *et al.*, 2018). Because EEG has a high temporal resolution and simplicity of measurement rather than fMRI, EEG-based applications are relatively suitable for diagnosis and treatment. Activation of theta power could be used for monitoring or neuro-feedback as a feature for craving state during CET.

In summary, we demonstrate that FG videos more strongly induce TP_PO_ than NFG videos, and increased TP_PO_ is a reliable feature indicating the craving state with novel experimental protocols unlike those of any other craving studies. This quantitative feature-based neuro-feedback or monitoring system could be practically applied to diagnosis and treatment targeting on IGD (e.g. CET). And many researchers for other addiction can also refer to the finding that favorite stimuli induce a higher level of craving than that induced by non-favorite stimuli.

## Supplementary Material

nsaa131_Supp
